# Targeting CDK9 for the Treatment of Glioblastoma

**DOI:** 10.3390/cancers13123039

**Published:** 2021-06-18

**Authors:** Alice Ranjan, Ying Pang, Madison Butler, Mythili Merchant, Olga Kim, Guangyang Yu, Yu-Ting Su, Mark R. Gilbert, David Levens, Jing Wu

**Affiliations:** 1 Neuro-Oncology Branch, Center for Cancer Research, National Cancer Institute, Bethesda, MD 20892, USA; alice.ranjan@nih.gov (A.R.); ying.pang@nih.gov (Y.P.); madisonkbutler@gmail.com (M.B.); mythili.merchant@nih.gov (M.M.); olga.kim@nih.gov (O.K.); guangyang.yu@nih.gov (G.Y.); filaminb@gmail.com (Y.-T.S.); mark.gilbert@nih.gov (M.R.G.); 2 Laboratory of Pathology, Center for Cancer Research, National Cancer Institute, Bethesda, MD 20892, USA; levensd@mail.nih.gov

**Keywords:** glioblastoma, CDK9 inhibitor, clinical trial

## Abstract

**Simple Summary:**

Inhibition of cyclin-dependent kinase 9 (CDK9) can impact multiple survival pathways in cancers and may be a promising therapeutic approach for glioblastoma, which is known to be highly resistant to treatments and thus challenging to treat. This review assesses the mechanisms by which CDK9 inhibition impacts cancer cell survival pathways in glioblastoma and other cancer types and presents results from clinical trials involving CDK9 inhibitors. A more thorough understanding of these mechanisms may lead to novel combination treatment strategies involving CDK9 inhibitors that can ultimately improve clinical outcomes for glioblastoma patients.

**Abstract:**

Glioblastoma is the most common and aggressive primary malignant brain tumor, and more than two-thirds of patients with glioblastoma die within two years of diagnosis. The challenges of treating this disease mainly include genetic and microenvironmental features that often render the tumor resistant to treatments. Despite extensive research efforts, only a small number of drugs tested in clinical trials have become therapies for patients. Targeting cyclin-dependent kinase 9 (CDK9) is an emerging therapeutic approach that has the potential to overcome the challenges in glioblastoma management. Here, we discuss how CDK9 inhibition can impact transcription, metabolism, DNA damage repair, epigenetics, and the immune response to facilitate an anti-tumor response. Moreover, we discuss small-molecule inhibitors of CDK9 in clinical trials and future perspectives on the use of CDK9 inhibitors in treating patients with glioblastoma.

## 1. Introduction

Gliomas are the most common primary malignant brain tumors in adults [1]. Glioblastoma accounts for the majority of gliomas (57.7%) and 48.6% of all primary malignant central nervous system (CNS) tumors and remains incurable [2]. In 2005, a seminal study in the field of neuro-oncology established the current standard of care for newly diagnosed glioblastoma, which involves surgery followed by radiation and concomitant temozolomide chemotherapy (TMZ) with adjuvant TMZ [3]. Despite this aggressive treatment regimen, only about 26% of patients survive two years after their initial diagnosis [3] and often experience disease relapse after 7 months of starting treatment [4]. Once glioblastoma recurs, the median progression-free survival is only about 9 weeks [5]. The high degree of disease recurrence and treatment resistance in glioblastoma patients underscores the need to identify novel therapies.

Despite extensive research efforts in the past decades, there has been limited advancement in identifying effective therapeutic approaches for glioblastoma, and few Food and Drug Administration (FDA) approved treatments are currently available [6]. The challenges to curing this disease are multifold. First, glioblastomas rarely show dependence on a single oncogene or tumor suppressor, which makes targeting single pathways ineffective in controlling the disease [7]. Therapies with the potential to modulate multiple cancer cell survival pathways are thus needed. Furthermore, while many molecular targets involved in the pathogenesis of the disease have been identified, some key targets are often considered undruggable [8]. These key targets include the amplification of genes encoding transcription factors like *MYC*, which are often difficult to directly inhibit [8]. Additionally, glioblastomas demonstrate high levels of intra- and inter-tumoral heterogeneity, with the landscape of heterogeneity changing as the tumor evolves during treatment [8,9]. In addition to these genetic features, certain physiological features impede the efficacy of treatments, especially immunotherapies, in glioblastomas. The blood-brain barrier (BBB) hinders the ability of many drugs to reach the tumor to any clinically meaningful degree [8]. Furthermore, glioblastomas contain an immunosuppressive tumor microenvironment (TME) due to the downregulation of chemokines that attract cytotoxic immune cells and the induction of chemokines that activate immunosuppressive cells and elicit T cell dysfunction [10,11]. Lastly, glioblastomas harbor self-renewing glioblastoma stem cells (GSCs) that promote intra-tumoral heterogeneity and contribute to resistance against surgery, radiation, and chemotherapy [12].

Given the rapid cellular proliferation and dependence on multiple survival pathways in glioblastomas, targeting cyclin-dependent kinases (CDKs) may be a promising strategy to overcome these challenges. CDKs are serine-threonine kinases that form heterodimers with specific types of cyclins and are generally categorized into two groups: those that regulate cell cycle progression (such as CDK1, CDK2, CDK4, and CDK6) and those that regulate transcription by RNA Polymerase II (RNA Pol II) (mainly CDK7, CDK8, and CDK9) [13]. Activation of CDKs and cyclins are tightly controlled in normal cells to maintain homeostasis [4]. Dysregulation of CDKs and cyclins results in abnormal cell proliferation and cancer progression, and thus, targeting CDKs has become an active area of research for both blood and solid tumors [4]. In fact, in 2015, the FDA approved the first CDK inhibitor—palbociclib (a dual CDK4/6 inhibitor)—to treat ER-positive and HER2-negative breast cancer [14]. While palbociclib monotherapy has not been an effective treatment for recurrent glioblastoma [15], two other CDK4/6 inhibitors, abemaciclib and ribociclib, are currently being investigated in phase I trials for adult and pediatric patients with glioblastoma or other brain tumors (NCT04074785, NCT03834740, NCT04238819, NCT02644460, NCT03434262) [16,17,18,19,20].

The idea that transcriptional programs are highly dysregulated in cancers and that cancers become over-dependent on certain factors that control gene expression has led to increased efforts to inhibit transcriptional CDKs, such as CDK9 [21]. Preclinical studies in several cancer types, including osteosarcoma, synovial sarcoma, endometrial cancer, and leukemia, have focused on targeting CDK9 due to its role in controlling transcription of super-enhancer driven oncogenes such as *MYC* and anti-apoptotic proteins such as myeloid-cell leukemia 1 (MCL-1), which maintain cancer cell survival [22,23,24,25,26]. Clinically, it has been observed that CDK9 is overexpressed in many cancer types, such as pancreatic cancer, osteosarcoma, synovial sarcoma, and endometrial cancer [23,24,25,27,28], and that a high CDK9 expression correlates with poor patient prognosis [23,24,25,27]. These trends have been observed in certain types of brain tumors as well. In medulloblastoma, CDK9 is highly expressed, and higher expression of CDK9 was shown to be correlated with poor patient prognosis [29]. Furthermore, pharmacological inhibition of CDK9 by LDC067 in medulloblastoma cells and by TG02 (also referred to as zotiraciclib) in meningioma cells was found to suppress cell growth [29,30]. In glioblastomas, CDK9 was also found to be highly expressed compared to non-tumor-containing brain samples [31]. Moreover, in patients with non-CpG island methylator phenotype (a subset of glioblastoma patients with poor survival outcomes), higher expression of CDK9 was found to correlate with worse clinical prognosis [31].

In this review, we discuss how targeting CDK9 may help overcome the challenges in treating glioblastomas by modulating not only transcription but also tumor cell metabolism, DNA damage repair, epigenetics, and the immune response. Furthermore, we discuss small-molecule inhibitors of CDK9 that have been or are currently being tested in clinical trials and future directions of targeting CDK9 for the management of glioblastoma.

## 2. CDK9: An Important Regulator of Transcription Elongation

**Figure 1:**
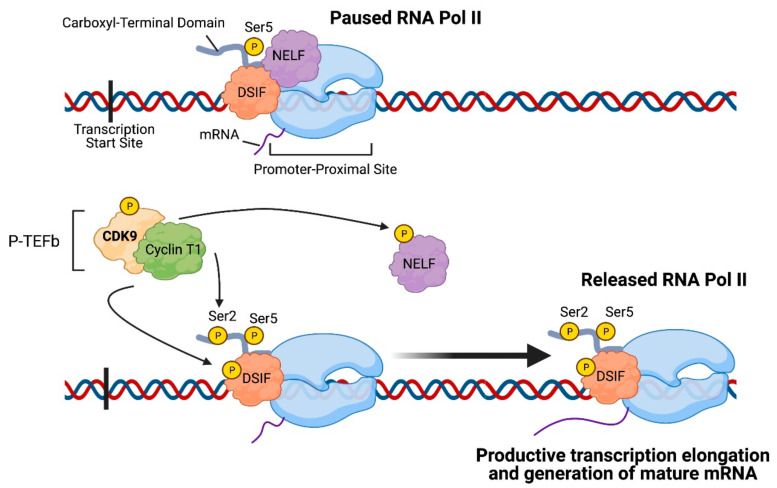
Role of CDK9 in transcription elongation: Positive transcription elongation factor b (P-TEFb), which is composed of cyclin-dependent kinase 9 (CDK9) and Cyclin T1, phosphorylates Serine 2 on the carboxyl-terminal domain of RNA Polymerase II (RNA Pol II) as well as negative elongation factor (NELF) and DRB-sensitivity-inducing factor (DSIF). Consequently, RNA Polymerase II is released from the promoter-proximal site and engages in productive transcription elongation and generation of mature mRNA. The image was created with BioRender.com (accessed on 18 April 2021).

CDK9 is broadly expressed in all types of human tissues and is present in two isoforms in mammalian cells: CDK9-49 and CDK9-55, which differ only by their molecular weight, but functionally are both able to associate with cyclins T1, T2A, T2B, or K (with CDK9 binding primarily to cyclin T1) [32]. The CDK9-cyclin T1 complex forms the positive transcription elongation factor b (P-TEFb), which plays a crucial role in regulating transcription elongation (Figure 1) [33]. Shortly after the initiation of transcription, RNA Pol II pauses at the promoter-proximal region, located 30–60 nucleotides downstream of the transcription start site [34]. This pausing of RNA Pol II serves as a quality control step to allow for 5′-capping and other modifications and is facilitated by promoter-associated transcription factors, negative elongation factor (NELF), and DRB-sensitivity-inducing factor (DSIF). For elongation to continue and for mature mRNA to be generated, the paused RNA Pol II must be released from the promoter-proximal site, and P-TEFb serves as a main regulator of this step. In order for P-TEFb to be fully activated, CDK9 is first phosphorylated by CDK7 at Threonine 186 [35], and subsequently, P-TEFb phosphorylates Serine 2 of RNA Pol II’s carboxyl-terminal domain (CTD), NELF, DSIF, and the CTD-linker of RNA Pol II in order to release RNA Pol II [36,37].

P-TEFb can exist in two other states in the cell—either reversibly bound in an inhibitory complex consisting of HEXIM1/2 and the small nuclear ribonucleoprotein (snRNP) 7SK or assembled with other transcription factors in an active super elongation complex (SEC) that interacts with RNA Pol II’s CTD [34]. The mechanism by which P-TEFb is released from the inhibitory complex and recruited to the promoter-proximal site is mediated by Jumonji Domain Containing 6 (JMJD6) and Bromodomain-containing protein 4 (BRD4), with JMJD6 binding directly with CDK9 and BRD4 with Cyclin T1 [36]. Notably, an shRNA loss-of-function screen demonstrated that glioblastoma cells in an orthotopic xenograft mouse model, but not in vitro, were dependent on JMJD6 and BRD4 for survival [38]. Furthermore, expression of JMJD6 was shown to increase with glioma grade and inhibiting JMJD6 extended survival of the glioma-bearing mice [38]. Additionally, a study demonstrated that two multi-CDK inhibitors, flavopiridol, and SNS-032, reduced levels of phosphorylated RNA Pol II in glioblastoma cells and disrupted anchorage-independent growth and cancer cell migration, two hallmarks of cancer cells [39,40]. These results reveal that glioblastomas are dependent on RNA Pol II pause-release and CDK9-containing complexes for their survival and present a rationale for further exploration of targeting CDK9 and its interacting factors as a therapeutic approach.

## 3. Impact of CDK9 Inhibition on Cancer Cells

In this section, we review studies in glioblastoma and other cancer types that demonstrate how CDK9 inhibition can modulate various cancer cell survival pathways to facilitate an anti-tumor response. Importantly, CDK9 inhibition does not specifically target different subsets of genes in different tumors. Rather, many of the pathways that are impacted by CDK9 inhibition in the various cancers reflect the different pre-established gene expression profiles of their tissues of origin.

### 3.1. Transcription

**Figure 2:**
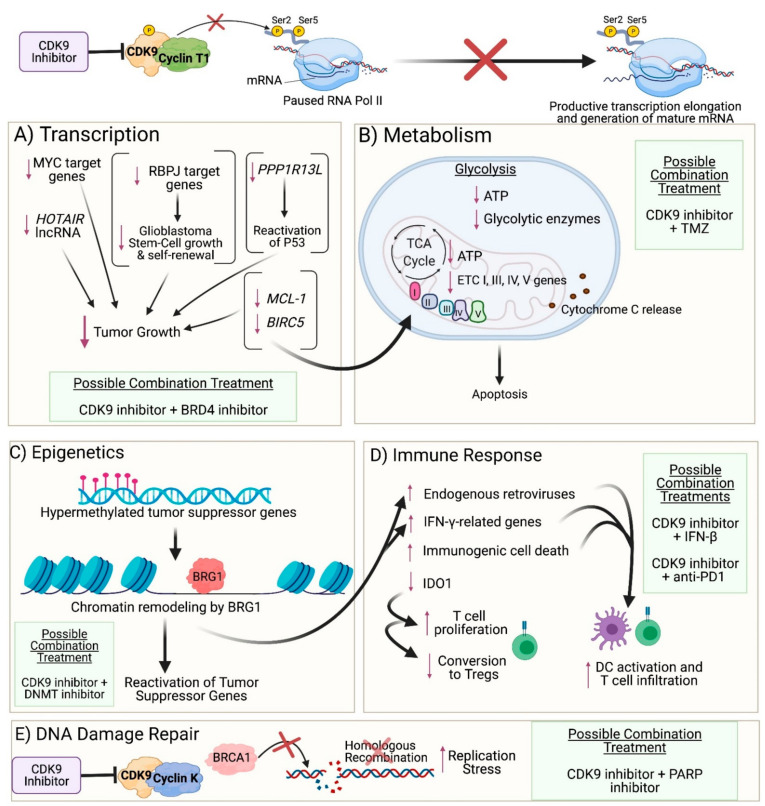
Impact of CDK9 inhibition on cancer cells: (**A**) Transcription: CDK9 inhibition prevents phosphorylation of Serine 2 on the carboxyl-terminal domain of RNA Polymerase II (RNA Pol II), thereby preventing productive transcription elongation of genes that are critical to the survival and proliferation of cancer cells, such as MYC target genes, *HOTAIR* lncRNA, *PPP1R13L*, RBPJ target genes, *MCL-1*, and *BIRC5*. (**B**) Metabolism: Reduced levels of anti-apoptotic proteins encoded by *MCL-1* and *BIRC5* result in mitochondrial dysfunction and damage as observed by the downregulated expression of genes involved in respiratory complexes I, III, IV, and V and the release of cytochrome *c* into the cytoplasm. Furthermore, CDK9 inhibition leads to reduced expression of glycolytic enzymes and ATP levels generated from both glycolysis and oxidative phosphorylation, ultimately leading to apoptosis. (**C**) Epigenetics: CDK9 inhibition enables recruitment of BRG1 to heterochromatin, where BRG1 remodels nucleosomes in order to facilitate transcription of genes, resulting in reactivation of tumor suppressor genes that were previously silenced by hypermethylation in their promoter region. (**D**) Immune Response: BRG1-mediated chromatin remodeling also results in reactivation of endogenous retroviruses and expression of IFN-γ-related genes. Moreover, CDK9 inhibition results in immunogenic tumor cell death and decreased expression of IDO1. Collectively, these events result in activation of dendritic cells (DCs) and proliferation and recruitment of effector T cells while reducing the conversion of T cells into regulatory T cells. (**E**) DNA Damage Repair: Inhibition of the CDK9-Cyclin K complex (but not the CDK9-Cyclin T1 complex) prevents CDK9-dependent BRCA1 recruitment to double-strand DNA breaks, thus precluding homologous recombination and resulting in increased replication stress. (**A**–**E**): Possible combination treatments involving CDK9 inhibitors are listed. The image was created with BioRender.com (accessed on 18 April 2021).

Given that CDK9 plays a crucial role in regulating transcription elongation, inhibiting CDK9 can reduce the transcription of genes necessary for maintaining cancer cell survival (Figure 2A). Su et al. demonstrated that zotiraciclib (a multi-kinase inhibitor that primarily targets CDK9) suppressed phosphorylation of CDK9 and RNA Pol II in glioblastoma cells, which resulted in decreased transcription of anti-apoptotic proteins such as MCL-1 and Survivin (encoded by *BIRC5*) and induced activation of caspase-3, resulting in cell apoptosis [35]. Furthermore, overexpression of a constitutively active CDK9 mutant rescued glioblastoma cells from zotiraciclib-induced cell death [35]. Le Rhun et al. note, however, that caspase activation is not essential for zotiraciclib-induced cell death [41]. The authors observed that glioblastoma cells co-exposed to zotiraciclib and a pan-caspase inhibitor demonstrated only moderately weakened zotiraciclib-induced cytotoxicity, and zotiraciclib still suppressed phosphorylation of RNA Pol II and depleted MCL-1 protein levels [41]. While the authors found that zotiraciclib-induced cell death occurs in a caspase-independent manner, they suggest that caspase inhibition may be involved in delaying cell death and that the proximate cause of death may be through zotiraciclib-induced metabolic alterations in glioblastoma cells, as demonstrated by Su et al. (see Section 3.2) [35,41].

CDK9 inhibition has also been reported as a useful strategy for treating cancers with MYC overexpression. MYC is of particular interest as a target in glioblastoma, given that it regulates about 15% of the entire genome, modulating cell proliferation, differentiation, survival, and apoptosis [42], and dysregulation in MYC signaling contributes to tumorigenesis [43]. However, MYC also maintains the proliferation of non-tumor cells and lacks effective binding pockets for small-molecule drugs, making its direct pharmacological inhibition challenging [42]. This underscores the need for methods that can indirectly inhibit MYC signaling, such as potentially targeting transcription of the *MYC* gene instead [42]. Importantly, transcription elongation of *MYC* is largely dependent on P-TEFb-mediated promoter-pause release [44]. Furthermore, MYC recruits P-TEFb to promoters to enhance transcription of its target genes, and MYC-overexpressing tumor cells are dependent on this activity [44]. A clinical trial for the highly selective CDK9 inhibitor KB-0742 was even launched recently in January 2021 to treat MYC-amplified cancers and is currently enrolling patients with advanced solid tumors or non-Hodgkin’s lymphoma [45]. Preclinically, for glioblastomas, zotiraciclib has been shown in one study to potently suppress the growth of MYC-overexpressing glioblastoma cells, and higher MYC expression was correlated with greater sensitivity to the drug [43]. However, another study demonstrated no correlation between gene silencing of *MYC* and sensitivity to zotiraciclib in glioblastoma cells [41]. Furthermore, zotiraciclib was shown to have varying effects on MYC transcript and protein expression levels: mRNA and protein expression decreased in some cell lines but increased in others following treatment and also varied based on the concentration of drug administered and time of exposure to the drug [41]. It is possible that CDK9 inhibitors may interfere more with MYC’s activity as a transcription factor than with transcription of the *MYC* gene. Furthermore, given that many pathways can activate MYC expression, it is possible that MYC expression can increase even with CDK9 inhibition due to other factors that possibly bypass promoter-proximal pausing, such as Aurora kinase A, which functions as a transactivating factor through its interaction with heterogeneous nuclear ribonucleoprotein K to activate MYC expression [46]. A study in HeLa cells similarly demonstrated an increase in MYC expression following treatment with i-CDK9, an inhibitor selective for CDK9, and the authors proposed that the increase in MYC expression may be part of a cellular compensatory mechanism to cope with CDK9 inhibition and to ensure maximal expression of important genes that are controlled by both MYC and CDK9 [47]. Moreover, BRD4 may be key to facilitating this compensatory mechanism –not only is BRD4 important in recruiting P-TEFb from the inhibitory 7SK snRNP/HEXIM1/2 complex to the promoter-proximal site, but it was shown in the study to use its C-terminal P-TEFb-interaction domain (PID) to directly increase CDK9′s catalytic activity and to render CDK9 more resistant to inhibition [47]. Furthermore, BRD4 inhibition was shown to reduce the interaction between BRD4 and CDK9 at the *MYC* locus and prevented the increase in MYC expression caused by i-CDK9 in HeLa, lung cancer, and melanoma cells [47]. Importantly, inhibition of both CDK9 and BRD4 exhibited a synergistic effect through the induction of apoptosis in HeLa and non-small cell lung cancer cells [47]. These findings thus suggest the value of targeting both CDK9 and BRD4 in MYC-overexpressing cells.

CDK9 inhibition has important implications not only for the oncogene *MYC* but for the tumor suppressor P53 as well. Loss of P53 function frequently occurs in the development of cancer either through mutations in the *TP53* gene or inhibition of the wild-type p53 protein by negative regulators [48]. The inhibitor of apoptosis-stimulating protein of p53 (iASPP), encoded by *PPP1R13L*, is one such negative regulator. Higher expression of the iASPP-SV isoform has been found to correlate with malignancy in gliomas, and glioblastomas may increase expression of iASPP-SV in order to promote tumor progression and prevent apoptosis [49]. Notably, glioma patients with high iASPP-SV expression experience lower overall survival and 5-year progression-free survival than patients with low iASPP-SV expression [49]. In a study conducted in a colon cancer cell line, the CDK9 inhibitors SNS-032, flavopiridol, and LDC067 were shown to downregulate transcription of iASPP, resulting in reactivation of the tumor-suppressive function of wild-type p53 [48]. The findings from this study may provide a promising CDK9-based strategy to counteract the pro-tumor effects of iASPP-SV in glioblastomas as well.

CDK9-mediated phosphorylation of RNA Pol II has also been demonstrated to be involved in a positive feedback loop that contributes to the upregulation and persistent expression of the long non-coding RNA HOX Transcript Antisense RNA (*HOTAIR*), which is overexpressed in multiple cancers and contributes to cancer progression [50]. *HOTAIR* has also been shown to promote malignant progression in gliomas and serves as a negative prognostic factor for survival of glioma patients [51]. A study demonstrated that pancreatic ductal adenocarcinoma, hepatocellular carcinoma, and colorectal cancer cells were unable to produce full-length, functional *HOTAIR* transcripts following CDK9 inhibition by LDC067 [50]. Targeting CDK9 may thus provide a mechanism to reduce *HOTAIR* expression in glioblastomas as well. Furthermore, it has been shown that BRD4 binds to the *HOTAIR* promoter and that treatment of glioblastoma cells with I-BET151 (an inhibitor against BRD4 and others in the bromodomain and extraterminal domain (BET) protein family) reduced levels of *HOTAIR* transcripts [52]. This provides yet another reason for the combined use of CDK9 and BRD4 inhibitors to synergistically inhibit proliferation of glioblastoma cells.

Additionally, CDK9 inhibition may provide a therapeutic approach to inhibit transcription of genes involved in maintaining GSCs. Efforts to target GSCs have often focused on inhibiting NOTCH since NOTCH signaling is critical in determining stem cell fate and cancer [53]. However, phase I trials investigating NOTCH antagonists in gliomas have shown limited efficacy [31]. An alternative solution may involve the recombination signal binding protein for immunoglobulin kappa J region (RBPJ), a transcription effector in NOTCH signaling that regulates a distinct transcription program compared to NOTCH [31]. Importantly, it was shown in a study that RBPJ relies on CDK9-mediated transcription elongation and that CDK9 inhibition results in decreased GSC growth and self-renewal [31]. Since GSCs are critical players in tumor formation and resistance to treatment, this finding supports the utility of targeting CDK9 to reduce treatment resistance caused by GSCs.

### 3.2. Metabolism

Targeting CDK9 has also been shown to induce metabolic stress in glioblastomas (Figure 2B). As previously discussed (see Section 3.1), zotiraciclib decreased transcription of the anti-apoptotic proteins MCL-1 and Survivin in glioblastoma cells, resulting in apoptosis [35]. Importantly, MCL-1 and Survivin also play vital roles in maintaining the function and integrity of the mitochondria. Zotiraciclib was shown to induce mitochondrial dysfunction in glioblastoma cells, as observed by the downregulation in expression of most genes involved in respiratory complexes I, III, IV, and V [35]. Moreover, zotiraciclib altered mitochondrial membrane potential and disrupted mitochondrial membrane integrity, as observed by the presence of dysmorphic mitochondria under electron microscopy and the release of cytochrome *c* into the cytoplasm by both Raman imaging and western blot [35]. Notably, zotiraciclib-induced mitochondrial damage was potentiated when TMZ treatment was included as well [35]. Zotiraciclib was also shown to suppress glycolysis, resulting in depleted intracellular ATP levels, and combination treatment of zotiraciclib and TMZ demonstrated a synergistic effect through further glycolytic suppression, as observed by the downregulation of the glycolytic enzymes Hexokinase 2 (HK2), Pyruvate Kinase isoform M2 (PKM2), and Lactate Dehydrogenase A (LDHA), which are usually highly expressed in glioblastomas [35,54,55,56]. Since HK2 and PKM2 promote tumor growth and GSC self-renewal [54,55] and glioblastomas are heavily reliant on glycolysis for energy production [57], targeting CDK9 provides a novel mechanism to exploit these metabolic vulnerabilities. Furthermore, since targeting CDK9 resulted in mitochondrial damage, glioblastoma cells are limited in their ability to compensate for energy production via oxidative phosphorylation. Inhibition of both energy production pathways thus increases the likelihood of cancer cell death.

### 3.3. DNA Damage Repair

Although CDK9 is often associated with transcription elongation, it also plays an important role in pathways that maintain genomic integrity [33]. Furthermore, Cyclin K, but not Cyclin T, is involved with CDK9 in these DNA damage repair processes: CDK9-Cyclin K interacts with DNA damage repair proteins such as ataxia telangiectasia and Rad3-related protein and accumulates on chromatin to limit the generation of single-stranded DNA resulting from DNA damage [33]. The CDK9-55 isoform, but not CDK9-42, has also been shown to associate with Ku70, a protein involved in double-strand DNA break (DSB) repair, and shRNA depletion of CDK9-55 in HeLa cells resulted in apoptosis and DSBs [58].

Given the importance of CDK9 and Cyclin K in DNA damage repair, studies in cancer models have focused on inhibiting CDK9 to generate increased replication stress and facilitate cancer cell death. In one study, osteosarcoma cells pre-treated with either the CDK9 inhibitor flavopiridol or 5,6-dichloro-1-β-D-ribofuranosylbenzimidazole demonstrated impaired cell cycle recovery following treatment with hydroxyurea [59]. Another study conducted in head and neck squamous cell carcinoma (HNSCC) cell lines examined the relationship between CDK9, DNA damage, and sensitivity to radiation [60]. siRNA knockdown of CDK9 resulted in significant induction of γH2AX (indicating DSBs), delayed cell cycle transition, and increased sensitivity to radiation. In contrast, overexpression of CDK9 enhanced the survival of HNSCC cells that were treated with radiation [60].

Glioblastomas demonstrate high levels of DNA replication, which predisposes them to significant replication stress [61]. BRCA1 is usually considered a tumor suppressor, but in glioblastomas, BRCA1 helps to mitigate replication stress and extend cancer cell survival [61]. High expression of BRCA1 has been shown to correlate with lower overall survival in glioblastoma patients [61], underscoring the need to investigate treatments that can dysregulate BRCA1 signaling. Importantly, CDK9 has been shown to modulate the recruitment of BRCA1 to DSBs and plays an important role in the BRCA1-mediated homologous recombination (HR) DNA repair process (Figure 2E) [62]. Cell survival assays performed in wild-type BRCA1 mammary gland carcinoma cells, for example, demonstrated that knockdown of CDK9 impaired recruitment of BRCA1 to DSBs and sensitized cells to radiation [62].

Tumors with mutations in BRCA1 are deficient in HR and are often vulnerable to treatment with inhibitors of Poly(ADP-ribose) polymerase (PARP), another enzyme involved in DNA repair processes [63]. Research has focused on expanding the benefit of PARP inhibitors to wild-type BRCA1 cancer cells by combining PARP inhibitors with other treatment options in order to achieve a synthetic lethal effect [63]. In one study, wild-type BRCA1 mammary gland carcinoma cells were treated with an shRNA targeting CDK9 and were found to be more sensitive to the combined treatment of radiation and Olaparib, a PARP inhibitor, than cells with intact CDK9 [62]. Another study in wild-type BRCA1 ovarian cancer cells demonstrated that the combined treatment of Olaparib and the CDK9 inhibitor CDKI-73 suppressed colony formation and induced apoptosis, and additionally reduced tumor growth in a xenograft mouse model [64]. Importantly, CDKI-73 was shown to downregulate BRCA1 expression, which contributed to increased sensitivity to Olaparib [64]. These studies present important implications for the treatment of glioblastomas: The majority of glioblastomas are reported to carry wild-type BRCA1 and are proficient in homologous recombination, and consequently, PARP inhibitors have shown limited efficacy [65]. Since BRCA1 is dependent on CDK9 for HR repair, CDK9 inhibition may provide a synthetic lethal mechanism to render glioblastomas more vulnerable to PARP inhibitors.

### 3.4. Epigenetics

Tumor suppressor genes (TSGs) in cancers are silenced via epigenetic modifications. Specifically, methylation in TSG promoters recruits repressor complexes that ultimately lead to the formation of heterochromatin [28]. Zhang et al. demonstrated that CDK9 maintains gene silencing in cancer cells by phosphorylating BRG1, a component of the SWI/SNF chromatin remodeling complex, preventing BRG1 from being recruited to the heterochromatin to move and restructure nucleosomes and mediate gene transcription [28]. Importantly, CDK9 inhibition was shown to dephosphorylate BRG1, enabling BRG1 to access and remodel the chromatin, leading to the reactivation of TSGs (Figure 2C) [28]. The authors also examined the anti-tumor effects of CDK9 inhibition in an ovarian cancer mouse model treated with SNS-032 and observed reactivation of ovarian-cancer specific hypermethylated TSGs, decreased tumor burden, and prolonged survival [28].

Glioblastomas exhibit frequent hypermethylation of TSGs, such as *RB1*, *EMP3*, *RASSF1A*, and *BLU* [66]. Moreover, one study reported that significant hypermethylation of the pro-apoptotic *CASP8* occurred during the progression from primary to recurrent glioblastoma, which possibly conferred a growth advantage to tumor cells remaining after radiation/chemotherapy treatment [67]. Targeting CDK9 may thus provide a mechanism to reactivate TSGs in glioblastomas and to counteract hypermethylation of factors that could render the tumor resistant to future treatments. Interestingly, Zhang et al. also demonstrated that the transcriptional profile induced by CDK9 inhibition was similar to the transcriptional profile induced by treatment with a DNA methyltransferase inhibitor (DNMTi), which demethylated the promoters of silenced TSGs, allowing for reactivation of these genes [28]. Combining CDK9 inhibition with DNMT inhibition may thus attain synergistic induction of TSGs and inhibition of tumor growth.

### 3.5. Immune Response

Given the immunosuppressive tumor microenvironment (TME) of glioblastomas, there is a need to identify better combination therapies that can improve the immune response against the tumor. Interferon (IFN)-β plays an immunosuppressive role in the TME by countering the pro-inflammatory effects of IFN-γ and preventing T cell trafficking into the CNS [10]. Interestingly, one study found that pre-exposure to IFN-β rendered glioblastoma cells more sensitive to subsequent treatment with zotiraciclib [68]. Specifically, combined treatment of IFN-β and zotiraciclib increased inhibition of cell growth compared to zotiraciclib treatment alone. The combined treatment also suppressed phosphorylation of RNA Pol II and reduced protein expression of CDK9 to a greater extent than zotiraciclib did alone, though how IFN-β mediates this synergistic effect remains to be explored [68]. On a separate but related note, a pharmacokinetic study of zotiraciclib, conducted as part of a phase I trial for patients with recurrent anaplastic astrocytoma and glioblastoma (NCT02942264) (see Section 5.2), revealed a significant increase in patient plasma concentrations of cytokines, including IP-10, at 24 h after an oral dose of zotiraciclib [69]. IP-10 has been reported to promote an anti-tumor immune response by attracting cytotoxic T and NK cells [10], though whether this occurs following zotiraciclib treatment remains to be determined. The induction of IP-10, along with the synergistic effect of IFN-β and zotiraciclib on glioblastoma cells, indicates the potential of zotiraciclib to counteract the immunosuppressive TME as one of the mechanisms of its anti-glioma effects.

The role of CDK9 inhibition in modulating the immune system (Figure 2D) has been further elucidated by Zhang et al. In one of their studies, colon cancer cells were treated with the CDK9 inhibitor HH1, and RNA sequencing of the cells identified upregulation of 326 immune-related genes [28]. Among these were endogenous retroviruses (ERVs), genetic elements originating from retroviruses that infected our ancestral germline and that are rarely expressed in healthy cells but can become expressed in cancer cells due to epigenetic dysregulation [70]. Importantly, the expression of ERVs may mimic a viral infection and induce IFN, thereby serving as potential tumor-associated antigens that can activate cytotoxic T cells [70]. Indeed, the 326-immune related genes from Zhang et al.’s study included genes in the IFN-γ pathway as well as the Major Histocompatibility Complex genes/Human Leukocyte Antigens HLA-A, HLA-B, and HLA-C [28]. Regarding glioblastoma, ERVs may serve as potential biomarkers for treatment given that a study characterizing the profile of ERVs in glioblastoma found 46 differentially expressed ERVs between glioblastoma and normal brain tissue, with 43 of those ERVs upregulated in glioblastoma [71].

Zhang et al. further demonstrated in an ovarian cancer mouse model that CDK9 inhibition via SNS-032 led to an increase in CD45+ immune cells, CD3+ T cells, and activated dendritic cells in the TME [28]. Furthermore, CDK9 inhibition sensitized the cells to anti-PD1 treatment, as demonstrated by an increased immune response following combination treatment [28].

Zhang et al. also observed in the TCGA database that colon cancer and melanoma patients with high expression of the same immune genes that were upregulated following CDK9 inhibition demonstrated significantly longer survival than patients with low expression of these genes [28]. This survival data, along with the prior results, provides evidence that targeting CDK9 may counteract the immunosuppressive TME and sensitize tumors to immune checkpoint inhibitors to yield potential clinical benefit.

CDK9 inhibition has also been shown to enhance the immune response against tumors by inducing immunogenic cell death (ICD) in tumor cells [72]. ICD occurs when dying cells release or express on their surface certain danger-associated molecular patterns that can activate an immune response [72]. Classic features of ICD include translocation of calreticulin (which is normally restricted to the endoplasmic reticulum (ER)) to the cell surface, the extracellular release of high mobility group box1 (HMGB1) and ATP, and activation of the Type I IFN pathway [72,73]. Altogether, ICD has the potential to induce dendritic cell (DC) activation and, ultimately, cross-presentation of antigens to cytotoxic T cells [73]. The multi-CDK inhibitor dinaciclib suppresses transcription of MCL-1 (which typically protects cells from ER stress) and was shown to elicit ICD in colon adenocarcinoma cells, as observed by the cell-surface expression of calreticulin and release of HMGB1 and ATP [72]. Furthermore, dinaciclib stimulated transient expression of Type I IFN response genes when administered as a single-agent treatment and in combination with anti-PD1 [72]. Importantly, combination treatment of dinaciclib and anti-PD1 resulted in increased DC activation and T cell infiltration and inhibition of tumor growth in a colon adenocarcinoma mouse model compared to dinaciclib and anti-PD1 single-agent treatments [72].

In addition to its ability to synergize with anti-PD1, dinaciclib can downregulate the expression of the immunosuppressive enzyme indoleamine 2,3-dioxygenase (IDO) in glioblastoma cells, as shown in a study by Riess et al. [74]. IDO is produced in response to IFN-γ and plays an important role in tryptophan metabolism by mediating the degradation of tryptophan and accumulation of kynurenine [11]. IDO-activity has been shown to inhibit T cell proliferation, promote T cell apoptosis, and induce regulatory T cells [11]. Glioblastomas exhibit high expression of IDO1, and increased IDO1 expression serves as a negative prognostic factor for patient survival [11]. Interestingly, Riess et al. also demonstrated that IDO1 expression increased in two glioblastoma cell lines following TMZ treatment and that IDO1 expression was reduced in TMZ-treated cells after additional treatment with dinaciclib [74]. This suggests the potential utility of combining CDK9 inhibitors with the standard treatment of TMZ in order to mitigate any immunosuppressive effects induced by TMZ via IDO1.

It is interesting to note that CDK9 inhibition can upregulate the expression of IFN-γ-stimulated genes [28] yet also suppress the activity of IDO1, an IFN-γ-stimulated gene [74]. CDK9 inhibition may serve then as a strategy to upregulate IFN-γ-stimulated genes that promote anti-tumor effects while mitigating immunosuppressive effects elicited by IDO1.

While the above studies highlight the advantages of inhibiting CDK9 in order to mediate a pro-inflammatory response against cancer cells, systemic blockade of CDK9 activity also has the potential to suppress the adaptive immune system. In fact, CDK9 inhibition has been investigated as an anti-inflammatory therapeutic approach for inflammatory conditions, such as arthritis [75], since CDK9 inhibition was shown to increase the percentage of regulatory T cells in spleens from arthritic mice [75]. Furthermore, flavopiridol inhibited the recruitment of NF-κB (a pro-inflammatory transcription factor that binds to P-TEFb to stimulate transcription elongation) in human endothelial cells [75]. This led to reduced ICAM-1 expression, which is important for recruiting lymphocytes to sites of inflammation [75]. In another study, zotiraciclib was shown to abrogate B cell receptor (BCR) signaling, though this provides a strategy for treating chronic lymphocytic leukemia (CLL) since leukemia cell survival is partly sustained through constitutive activation of BCR signaling [76]. Furthermore, CDK9 inhibition has the potential to adversely affect T cell activation. The dual CDC7/CDK9 inhibitor PHA-767491/NMS-1116354 was shown to affect signal transduction downstream of the T cell receptor (TCR) by inhibiting Erk phosphorylation, which is important for T cell activation and degrading the p105 isoform of NF-κB, which is important for regulating T cell homeostasis [77]. The inhibitory effects of CDK9 on T cell signaling and function have been speculated to contribute to the adverse immune-related symptoms that patients receiving CDK inhibitors in clinical trials may experience (see Section 4) [77]. Further studies focusing on the optimal timing and dosage that allows for an immunogenic response against tumor cells without detrimental effects on T cell proliferation and effector functions are warranted.

## 4. CDK9 Inhibitors in Cancer Clinical Trials

CDK9 can be directly blocked by small-molecule inhibitors at its ATP-binding site [32]. Several inhibitors have been or are currently being tested in clinical trials for the treatment of various cancers [22] and are listed in Table 1 and summarized here. The majority of these inhibitors are potent against CDK9, with 50% inhibitory concentration (IC_50_) values in the nanomolar (nM) range [78]. However, they are also nonselective, and target other CDKs (often at IC_50_ values in the nM range as well) since the ATP-binding pocket is conserved in the entire CDK family [32,78]. Moreover, inhibitors may target kinases other than CDKs, resulting in some adverse effects [78]. This has limited the clinical utility of some inhibitors, resulting in termination from clinical trials. For example, ZK-304709, an inhibitor of CDKs 1, 2, 4, 7, and 9 as well as Vascular Endothelial Growth Factor Receptor (VEGFR)-1, 2, and 3, Platelet-derived Growth Factor Receptor (PDGFR)-β, and FMS-like Tyrosine Kinase (FLT)-3 [78,79], was prematurely terminated in a phase I study for patients with advanced solid tumors due to severe adverse effects of nausea and vomiting [80]. The investigation of SNS-032, which primarily targets CDKs 2, 7, and 9 [81], in a phase I study for patients with metastatic refractory solid tumors was similarly discontinued, in part because of toxicity issues, since all patients reported side effects, such as fatigue, nausea, diarrhea, and abdominal pain [81].

Some inhibitors have shown encouraging results in some of their clinical trials (while still demonstrating adverse outcomes in other trials) and have garnered orphan drug status by the FDA. One such drug is flavopiridol/alvocidib, which inhibits CDKs 1, 2, 4, 5, 6, 7, and 9 and Glycogen Synthase Kinase (GSK)-3β, though it is most potent against CDK9 with an IC_50_ of 3nM [78]. In 1994, flavopiridol became the first CDK inhibitor to enter clinical trials [22] and has now become the most frequently investigated CDK9 inhibitor in clinical trials [32]. A phase II study of flavopiridol in combination with cytarabine and mitoxantrone for patients with acute myeloid leukemia (AML) demonstrated 58% complete response [32], and in 2014, flavopiridol was granted orphan drug designation by the FDA for AML [77]. Despite this success, two other phase II trials testing flavopiridol (in primary peritoneal cancer and CLL, respectively, as listed in Table 1) reported only a 2% complete response to treatment [32]. Furthermore, while Table 1 provides only a sampling of phase I trials for flavopiridol, over half of 12 phase I trials reported no complete response to the drug [32,77,78] and involved adverse effects such as neutropenia, thrombocytopenia, and fatigue [32]. Dinaciclib/SCH-727965 is another multi-CDK inhibitor demonstrating high potency against CDKs 1, 2, 5, and 9, with IC_50_ values of 3 nM, 1 nM, 1 nM, and 4 nM, respectively [78]. Dinaciclib has been involved in various phase I and II trials, with similar reports of neutropenia and leukopenia, and was granted orphan drug status by the FDA in 2011 for CLL [77]. Zotiraciclib/TG02/SB1317 is a multi-kinase inhibitor demonstrating good penetration of the BBB based on preclinical studies using orthotopic glioblastoma mouse models [35]. While it primarily targets CDK9 (with an IC_50_ value of 3 nM) [78], it also inhibits CDKs 1, 2, and 7, Janus Kinase 2 (JAK2), and FLT3 [26]. Dose-limiting toxicities of zotiraciclib in a phase I trial for patients with recurrent anaplastic astrocytoma and glioblastoma included neutropenia as well, but the drug demonstrated an overall tolerable toxicity profile (see Section 5.3) and was granted orphan drug designation by the FDA in 2019 for treatment of gliomas [69].

## 5. Targeting CDK9 in Clinical Trials for Gliomas

### 5.1. Zotiraciclib as a Promising CDK9 Inhibitor for Treating Glioblastoma

To our knowledge, zotiraciclib is the primary CDK9 inhibitor being investigated in clinical trials focused specifically on gliomas. Abemaciclib, a CDK4/6 inhibitor that is currently being tested in phase I trials for adult and pediatric patients with glioblastoma or other brain tumors (see Introduction), has also been reported to demonstrate potent inhibition against CDK9 and more efficient BBB penetration compared to ribociclib and palbociclib, two other CDK4/6 inhibitors in phase I trials [85,86,87]. However, studies have focused mostly on abemaciclib’s inhibition of CDKs 4 and 6.

As previously discussed in this review (see Section 3.1, Section 3.2, Section 3.4 and Section 3.5), preclinical studies in glioblastoma cell lines and mouse models have demonstrated the ability of zotiraciclib to inhibit tumor growth via multiple mechanisms. Importantly, an additional study demonstrated that glioblastoma cells did not acquire resistance after repeated exposure to zotiraciclib; in fact, repeated exposure reduced cell growth in one cell line, induced senescence in a second cell line, and increased sensitivity to the drug in other cells [41]. Single-agent treatment of zotiraciclib was also shown to yield modest survival benefit in two mouse models that used human glioma cell lines [41], while another study found that zotiraciclib extended survival in a mouse model that used a mouse glioma cell line only when combined with TMZ [35]. These findings have led to the launching of three clinical trials investigating zotiraciclib in glioblastoma patients, as reviewed below.

### 5.2. Clinical Trials Investigating Zotiraciclib in Recurrent and Newly Diagnosed Brain Tumors

Based on the synergistic effects of zotiraciclib and TMZ demonstrated in the preclinical studies, a phase I/II clinical trial of zotiraciclib plus dose-dense or metronomic TMZ in patients with recurrent glioblastoma and anaplastic astrocytoma (AA) (NCT02942264) was launched [88], and the phase I part has recently been completed [69]. In this two-stage phase I study, the maximum tolerated dose (MTD) of zotiraciclib combined with either dose-dense TMZ (125 mg/m^2^ × 7 days on/7 days off) or metronomic TMZ (50 mg/m^2^ daily) was first determined [69]. This was followed by a randomized cohort expansion to compare the progression-free survival rate at 4 months (PSF4) between the two arms to determine a TMZ schedule to combine with zotiraciclib at the MTD [69]. The MTD of zotiraciclib in both arms was found to be 250 mg, and dose-dense TMZ was selected based on a favorable PFS4 of 40% compared to metronomic TMZ (25%) [69]. Zotiraciclib was found to be safe and tolerable, though patients experienced some side effects (see Section 5.3). A randomized phase II trial to determine the efficacy of zotiraciclib and dose-dense TMZ has been planned.

Zotiraciclib is also under investigation as a monotherapy for recurrent high-grade gliomas in a phase I clinical trial (NCT03904628) [89]. In this trial, adult patients with recurrent glioblastoma or AA who failed TMZ treatment in the past are potential candidates [89]. Eligible patients will be given zotiraciclib twice a week at 150 mg–250 mg, and the MTD of zotiraciclib will be determined once the study is completed [89].

A phase Ib study evaluating the safety and progression-free survival at 6 months (PFS6) of zotiraciclib in newly diagnosed elderly patients or adults with recurrent glioblastoma or AA is also ongoing (NCT03224104) [90]. NCT03224104 includes three experimental arms: In Group A, elderly patients with IDH1 wild-type and MGMT unmethylated AA or glioblastoma will receive zotiraciclib and radiation therapy [90]. In Group B, elderly patients with IDH1 wild-type and MGMT methylated AA or glioblastoma will receive zotiraciclib and TMZ [90]. Two dose levels of zotiraciclib will be tested in Groups A and B [90]. In Group C, adult patients with IDH1 wild-type AA or glioblastoma will receive zotiraciclib at recurrence after receiving radiation plus concurrent TMZ followed by adjuvant TMZ [90]. A recommended phase II dose of zotiraciclib will be determined in Groups A and B while PFS6 will be determined in Group C once the study is completed [90].

### 5.3. Toxicity Profile of Zotiraciclib

Preclinical studies have evaluated the cytotoxicity of zotiraciclib in glioblastoma cells as well as non-tumor cells [35]. While zotiraciclib induced cytotoxicity and reduced long-term survival of glioblastoma cells, zotiraciclib-treated astrocytes demonstrated no cytotoxicity—no significant mitochondrial dysfunction, apoptosis, or inhibition of cell survival and proliferation—compared to glioblastoma cells [35]. Incidentally, flavopiridol and SNS-032 have also been shown to induce cytotoxicity in glioblastoma cells but not in primary neurons [39]. This finding, coupled with the lack of cytotoxic effects in astrocytes, suggests the potential clinical benefit of using CKD9 inhibitors in brain tumors without causing significant toxicities in the CNS.

The cytotoxic effect of zotiraciclib on endothelial and epithelial cells has also been examined. No cytotoxicity was observed in arterial endothelial cells [35]. Single-agent treatment of zotiraciclib and combined treatment of zotiraciclib and TMZ were shown to induce cytotoxicity in microvascular endothelial cells compared to endothelial cells from large vessels but to a much lesser degree than in glioblastoma cells [35]. Human intestinal epithelial cells treated with zotiraciclib exhibited cytotoxicity, though to a lesser extent compared to glioblastoma cells [35]. This finding correlates with observations from a study conducted in a mouse intracranial xenograft model, which investigated the synergistic anti-tumor effects of zotiraciclib and TMZ combined compared to single-agent treatment of zotiraciclib or TMZ [35]. It was noted that several mice treated with zotiraciclib alone and several mice treated with zotiraciclib and TMZ combined suffered from gastrointestinal toxicities such as diarrhea [35]. However, the study found overall that combining zotiraciclib and TMZ reduced tumor growth by 25% and significantly prolonged survival while single-agent treatment of zotiraciclib or TMZ resulted in no reduction in tumor growth and no survival benefit [35].

In the phase I trial (NCT02942264) testing zotiraciclib plus TMZ in patients with recurrent glioblastoma and AA, dose-limiting toxicities of zotiraciclib included diarrhea (thus correlating with the preclinical findings), neutropenia, elevated liver enzymes, and fatigue [69]. For all dose levels of zotiraciclib and the different TMZ dosing schedules, common non-hematologic treatment-related adverse events (AEs) included elevated enzymes, diarrhea, fatigue, and nausea, occurring at grades 1–2. The most common non-hematologic AE occurring at grades 3–4 was the elevation of alanine aminotransferase, as observed in 20.8% of patients, with one case attributed to zotiraciclib alone [69]. 9.4% of patients developed grade 3 fatigue, with 3 out of 5 cases most likely resulting from zotiraciclib alone, and 5.6% of patients experienced grade 3 diarrhea, due to zotiraciclib alone [69]. The majority of hematologic AEs were grades 1–2, though 24.5% of patients experienced grade 4 neutropenia [69]. In particular, an unusual pattern of neutropenia was observed: 9 patients recovered to grade 2 or less in 3 days, and 3 patients recovered to grade 3 in 3 days [69]. To better understand the consequences of the observed zotiraciclib-induced neutropenia, in-depth neutrophil analyses and a pharmacokinetic study of zotiraciclib were performed simultaneously. Results demonstrated that the neutropenia is a significant but transient phenomenon that does not compromise patient safety [69].

## 6. Conclusions and Future Directions

Glioblastomas have remained incurable due to challenges caused by the genetic and microenvironmental characteristics of the disease. Targeting CDK9 may be a promising approach due to its ability to modulate various cellular mechanisms to initiate an anti-tumor response, as discussed in this review. However, many CDK9 inhibitors that have been or are currently being investigated in clinical trials are nonselective and have a narrow therapeutic window [78], underscoring the need to develop drugs with greater selectivity. Furthermore, since it is possible for drugs to be used at lower concentrations when combined and demonstrate synergistically lethal effects on cancer cells [47], it will be instructive to investigate combination treatments of CDK9 inhibitors with agents such as TMZ, BRD4 inhibitors, DNMT inhibitors, PARP inhibitors, IFN-β, and anti-PD1 in order to mitigate off-target effects while enhancing anti-tumor effects. Continued emphasis on precision medicine to predict drug metabolism and allow for personalized drug dosing will also be integral to reducing drug-related toxicities. For example, the pharmacokinetic and pharmacogenomic studies of zotiraciclib conducted as part of the phase I trial for patients with recurrent glioblastoma and AA (NCT02942264) identified a single nucleotide polymorphism in the gene encoding CYP1A2 (*CYP1A2_5347T>C*, N541N, *rs2470890*), an enzyme that metabolizes zotiraciclib, which significantly altered the pharmacokinetics of the drug in a certain cohort of patients [69]. As part of precision medicine, it will also be necessary to identify specific biomarkers in glioblastoma that can predict treatment response or resistance to CDK9 inhibition, and once determined, these biomarkers can be used to identify subsets of patients that will benefit most from the treatment. Lastly, further prospective clinical trials will be needed to assess the long-term impact of CDK9 inhibitors on patient outcomes.

## Figures and Tables

**Table 1:** Overview of CDK9 inhibitors in cancer clinical trials.

No.	Inhibitor Name	Targets (Including CDK9)	Investigated in Clinical Trial for Gliomas	Clinical Trial (with NCT Identifier from ClinicalTrials.gov accessed on 16 April 2021), If Applicable)	Phase	Trial Status	Cancer Type
1	AT7519	CDK1, CDK2, CDK4, CDK5, CDK6, CDK9 [78]	N/A	NCT01652144: A Phase II Study of AT7519M, a CDK Inhibitor, in Patients with Relapsed Mantle Cell Lymphoma	II	Completed	Mantle Cell Lymphoma
				NCT01627054: A Phase II Study of AT7519M, a CDK Inhibitor, in Patients with Relapsed and/or Refractory Chronic Lymphocytic Leukemia	II	Completed	Refractory Chronic Lymphocytic Leukemia
				NCT01183949: Effect of AT7519M Alone and AT7519M Plus Bortezomib in Patients with Previously Treated Multiple Myeloma	I/II	Completed	Multiple Myeloma
				NCT02503709: Onalespib and CDKI AT7519 in Treating Patients with Solid Tumors That Are Metastatic or Cannot Be Removed by Surgery	I	Active, not recruiting	Advanced Malignant Solid Neoplasms, Metastatic Malignant Solid Neoplasms, and Unresectable Solid Neoplasms
				NCT00390117: AT7519M in Treating Patients with Advanced or Metastatic Solid Tumors or Refractory Non-Hodgkin’s Lymphoma	I	Completed	Advanced or Metastatic Solid Tumors and Refractory Non-Hodgkin’s Lymphoma
2	Atuveciclib/BAY-1143572	CDK2 and CDK9 [78]	N/A	NCT02345382: Phase I Dose Escalation of BAY1143572 in Subjects with Acute Leukemia	I	Completed	Acute Leukemia
				NCT01938638: Open Label Phase I Dose Escalation Study with BAY1143572 in Patients with Advanced Cancer	I	Completed	Advanced Cancers
3	AZD-4573	CDK1 and CDK9 [78]	N/A	NCT03263637: Study to Assess Safety, Tolerability, Pharmacokinetics and Antitumor Activity of AZD4573 in Relapsed/Refractory Haematological Malignancies	I	Recruiting	Relapsed/Refractory Haematological Malignancies
4	BAY-1251152	CDK9 [78]	N/A	NCT02745743: Phase I Trial of BAY1251152 for Advanced Blood Cancers	I	Completed	Advanced Blood Cancers
				NCT02635672: Phase I Dose Escalation Study for BAY 1251152 in Patients with Advanced Cancer	I	Active, not recruiting	Advanced Cancers
5	BTX-A51	CDK7 and CDK9 [78]	N/A	NCT04243785: A Study of BTX-A51 in People with Relapsed or Refractory Acute Myeloid Leukemia or High-Risk Myelodysplastic Syndrome	I	Recruiting	Acute Myeloid Leukemiad and Myelodysplastic Syndrome
6	CYC065/Fadraciclib	CDK2, CDK5, CDK7, CDK9 [78]	N/A	NCT03739554: CYC065 CDK Inhibitor and Venetoclax Study in Relapsed/Refractory CLL	I	Recruiting	Relapsed or Refractory Chronic Lymphocytic Leukemia
				NCT04017546: CYC065 CDK Inhibitor and Venetoclax Study in Relapsed/Refractory AML or MDS	I	Recruiting	Acute Myeloid Leukemia and Myelodysplastic Syndromes
7	Dinaciclib/SCH-727965	CDK1, CDK2, CDK5, CDK9 [78]	N/A	NCT00732810: SCH-727965 in Patients with Advanced Breast and Lung Cancers	II	Completed	Breast Neoplasms and Non-Small-Cell Lung Cancer
				NCT00798213: SCH-727965 in Patients with Acute Myelogenous Leukemia and Acute Lymphoblastic Leukemia	II	Terminated	Acute Myelogenous Leukemia and Acute Lymphoblastic Leukemia
				NCT00871663: Phase 1 Weekly Dosing of SCH 727965 in Patients with Advanced Cancer	II	Completed	Solid Tumors, Non-Hodgkin Lymphoma, Multiple Myeloma, and Chronic Lymphocytic Leukemia
				NCT01096342: Dinaciclib in Treating Patients with Relapsed or Refractory Multiple Myeloma	II	Completed	Refractory Multiple Myeloma
				NCT01650727: A Study of Dinaciclib in Combination with Rituximab in Participants with Chronic Lymphocytic Leukemia and Small Lymphocytic Lymphoma	I	Completed	Chronic Lymphocytic Leukemia and Small Lymphocytic Lymphoma
8	Flavopiridol/Alvocidib	CDK1, CDK2, CDK4, CDK5, CDK6, CDK7, CDK9, GSK3β [32,77,78]	N/A	NCT00083122: Cisplatin and Flavopiridol in Treating Patients with Advanced Ovarian Epithelial Cancer or Primary Peritoneal Cancer	II	Completed	Ovarian Epithelial Cancer and Primary Peritoneal Cancer
				NCT00407966: Alvocidib, Cytarabine, and Mitoxantrone in Treating Patients with Newly Diagnosed Acute Myeloid Leukemia	II	Completed	Acute Myeloid Leukemia
				NCT00464633: Alvocidib in Patients with Previously Treated Chronic Lymphocytic Leukemia or Prolymphocytic Leukemia Arising From Chronic Lymphocytic Leukemia (CLL)	II	Completed	Chronic Lymphocytic Leukemia, Prolymphocytic Leukemia arising from Chronic Lymphocytic Leukemia
				NCT03593915: Study of Alvocidib Plus Decitabine or Azacitidine in Patients with MDS	Ib/II	Active, not recruiting	Myelodysplastic Syndromes
				NCT00112723: Flavopiridol in Treating Patients with Relapsed or Refractory Lymphoma or Multiple Myeloma	I/II	Terminated	Lymphoma and Multiple Myeloma
				NCT00112684: Alvocidib in Treating Patients with Locally Advanced or Metastatic Solid Tumors	I	Terminated	Advanced or Metastatic Solid Tumors
				NCT00082784: Bortezomib and Flavopiridol in Treating Patients with Recurrent or Refractory Indolent B-Cell Neoplasms	I	Completed	B-Cell Neoplasms
				NCT00470197: Flavopiridol, Cytarabine, and Mitoxantrone in Treating Patients with Relapsed or Refractory Acute Leukemia	I	Completed	Relapsed or Refractory Acute Leukemia
9	KB-0742	CDK9 [82]	N/A	NCT04718675: A Dose Escalation and Cohort Expansion Study of KB-0742 in Participants with Relapsed or Refractory Solid Tumors or Non-Hodgkin’s Lymphoma	I	Recruiting	Relapsed or Refractory Solid Tumors and Non-Hodgkin’s Lymphoma
10	NMS-1116354	CDK9, CDC7 [77,83]	N/A	NCT01016327: Study of NMS-1116354 in Solid Tumors	I	Terminated (Discontinuation of clinical investigation of drug)	Advanced Solid Tumors
				NCT01092052: Study of NMS-1116354 in Advanced/Metastatic Solid Tumors	I	Terminated (Discontinuation of clinical investigation of drug)	Advanced/Metastatic Solid Tumors
11	RGB-286638	CDK1, CDK2, CDK3, CDK4, CDK5, and CDK9 (less active against CDK6 and CDK7) [84]	N/A	NCT01168882: Safety and Tolerability of RGB-286638 in Patients with Selected, Relapsed or Refractory Hematological Malignancies	I	withdrawn	Hematological Malignancies
12	Riviciclib/P-276-00	CDK1, CDK2, CDK4, CDK6, CDK9 [78]	N/A	NCT00824343: A Phase II Clinical Trial to Study the Efficacy and Safety of a New Drug P276-00 in Treatment of Recurrent and/or Locally Advanced Head and Neck Cancer (MONARCH)	II	Completed	Advanced Head and Neck Cancer
				NCT00843050: A Phase II Study to Evaluate Efficacy and Safety of P276-00 in Relapsed and/or Refractory Mantle Cell Lymphoma	II	Terminated (based on interim results; no major safety or tolerability concerns)	Mantle Cell Lymphoma
				NCT00898287: Safety and Efficacy Study of P276-00 in Combination with Gemcitabine in Patients with Advanced Pancreatic Cancer (SAVIOR)	I/II	Completed	Pancreatic Cancer
				NCT00899054: Safety and Efficacy Study of P276-00 in Combination with Radiation in Subjects with Advanced Head and Neck Cancer (SPARK)	I/II	Completed	Squamous Cell Carcinoma of Head and Neck
				NCT00882063: Study To Evaluate Safety and Efficacy of P276-00 in Subjects with Refractory Multiple Myeloma	I/II	Completed	Refractory Multiple Myeloma
				NCT01333137: A Clinical Trial Comparing Gemcitabine and Carboplatin with and without P276-00 in Subjects with Metastatic Triple Negative Breast Cancer, with a Run-in of Escalating Dose of P276-00 Added to Gemcitabine and Carboplatin	I	Terminated	Metastatic Triple Negative Breast Cancer
13	Roniciclib/BAY-1000394	CDK1, CDK2, CDK4, CDK5, CDK7, CDK9 [78]	N/A	NCT02656849: BAY 1000394 for MCL-1-, MYC-, and CCNE1-Amplified Tumors	II	withdrawn (Development of BAY1000394 has been terminated by Bayer)	Solid Tumors
				NCT02161419: RONICICLIB/Placebo in Combination with Chemotherapy in Small Cell Lung Cancer (CONCEPT-SCLC)	II	Terminated	Small Cell Lung Carcinoma
				NCT02522910: An Open-label Phase Ib/II Study of BAY 1000394 (Roniciclib) in Combination with Docetaxel in Second- or Third-line Treatment of Patients with Advanced Non-small Cell Lung Cancer (NSCLC)	Ib/II	withdrawn	Non-Small Cell Lung Cancer
				NCT01573338: Clinical Study to Evaluate the Maximum Tolerated Dose of BAY1000394 When Given Together with Chemotherapy and the Effectiveness of This Combination Treatment in Shrinking a Specific Type of Lung Tumors (Small Cell Lung Cancer)	I/II	Terminated	Small Cell Lung Cancer
				NCT01188252: Clinical Study to Evaluate the Maximum Tolerated Dose of BAY1000394 Given in a 3 Days on/4 Days Off Schedule in Subjects with Advanced Malignancies	I	Completed	Advanced Malignancies
14	Roscovitine/Seliciclib/CYC202	CDK2, CDK7, CDK9, DIRK1A, ERK1 [32,78]	N/A	NCT00372073: Efficacy Study of Oral Seliciclib to Treat Non-Small Cell Lung Cancer	II	Terminated	Non-Small Cell Lung Cancer
				NCT01333423: Maximum Tolerated Dose (MTD) of Liposomal Doxorubicin in Combination with Seliciclib for Patients with Metastatic Triple Negative Breast Cancer (TNBC)	I	withdrawn	Metastatic Triple Negative Breast Cancer
15	SNS-032	CDK1, CDK2, CDK5, CDK7, CDK9 [78]	N/A	NCT00292864: Safety Assessment of One-hour Infusions of SNS-032 for the Treatment of Select Advanced Solid Tumors	I	Terminated (based on report published by study investigators) [81]	Metastatic Refractory Solid Tumors
				NCT00446342: Study of Intravenously Administered SNS-032 in Patients with Advanced B-lymphoid Malignancies	I	Completed	B-lymphoid Malignancies, Chronic Lymphocytic Leukemia and Mantle Cell Lymphoma, and Multiple Myeloma
16	TP-1287	CDK1, CDK2, CDK4, CDK6, CDK7, CDK9 [78]	N/A	NCT03604783: Phase I, First-in-human Study of Oral TP-1287 in Patients with Advanced Solid Tumors	I	Recruiting	Advanced Solid Tumors
17	Voruciclib/P-1446	CDK1, CDK2, CDK4, CDK5, CDK6, CDK8, CDK9 [78]	N/A	NCT03547115: A Phase 1 Study of Voruciclib in Subjects with B-Cell Malignancies or AML	I	Recruiting	Follicular Lymphoma, Mantle Cell Lymphoma, Marginal Zone Lymphoma, Small Lymphocytic Lymphoma, Chronic Lymphocytic Leukemia, and Diffuse Large B-cell Lymphoma, Acute Myeloid Leukemia
18	ZK-304709	CDK1, CDK2, CDK4, CDK7, CDK9, VEGFR1, VEGFR2, VEGFR3, PDGFRβ, FLT3 [78,79]	N/A	(NCT Indicator N/A): A Phase I Dose Escalation Study of the Pharmacokinetics and Tolerability of ZK-304709, an Oral Multi-Targeted Growth Inhibitor (MTGI^™^), in Patients with Advanced Solid Tumors [80]	I	Terminated	Advanced Solid Tumors
19	Zotiraciclib/TG02/SB1317	CDK1, CDK2, CDK5, CDK7, CDK9, JAK2, FLT3 [26]	Yes	NCT02942264: Zotiraciclib (TG02) Plus Dose-Dense or Metronomic Temozolomide Followed by Randomized Phase II Trial of Zotiraciclib (TG02) Plus Temozolomide Versus Temozolomide Alone in Adults with Recurrent Anaplastic Astrocytoma and Glioblastoma	I/II	Phase I Completed	Recurrent Anaplastic Astrocytoma and Glioblastoma
				NCT03224104: Study of TG02 in Elderly Newly Diagnosed or Adult Relapsed Patients with Anaplastic Astrocytoma or Glioblastoma (STEAM)	I	Recruiting	Anaplastic Astrocytoma and Glioblastoma
				NCT01204164: Phase I Clinical Study of Oral TG02 Capsule in the Treatment of Recurrent/Progressive High-grade Glioma Patients	I	Recruiting	Recurrent/Progressive High-Grade Glioma
				NCT03738111: Study of TG02 Citrate in Patients with Advanced Hepatocellular Carcinoma	I	withdrawn	Advanced Hepatocellular Carcinoma
				NCT02933944: Exploratory Study of TG02-treatment as Monotherapy or in Combination with Pembrolizumab to Assess Safety and Immune Activation in Patients with Locally Advanced Primary and Recurrent Oncogenic RAS Exon 2 Mutant Colorectal Cancer	I	Terminated	Colorectal Cancer
				NCT01204164: Phase 1 Study of TG02 Citrate in Patients with Advanced Hematological Malignancies (TG02-101)	I	Completed	Advanced Hematological Malignancies
				NCT01699152: Phase 1 Study of TG02 Citrate in Patients with Chronic Lymphocytic Leukemia and Small Lymphocytic Lymphoma	I	Completed	Chronic Lymphocytic Leukemia and Small Lymphocytic Lymphoma
